# Increased risk of peripheral arterial occlusive disease in patients with Bell's palsy using population data

**DOI:** 10.1371/journal.pone.0188982

**Published:** 2017-12-07

**Authors:** Li-Syue Liou, Chih-Ya Chang, Hsuan-Ju Chen, Chun-Hung Tseng, Cheng-Yu Chen, Fung-Chang Sung

**Affiliations:** 1 Department of Family Medicine, Hualien Tzu Chi Hospital, Buddhist Tzu Chi Medical Foundation, Hualien City, Hualien County, Taiwan, ROC; 2 Department of Family Medicine, Taipei Tzu Chi Hospital, Buddhist Tzu Chi Medical Foundation, Xindian Dist., New Taipei City, Taiwan(R.O.C); 3 Department of Family and Community Medicine, Tri-Service General Hospital, Neihu District, Taipei City, Taiwan(R.O.C.); 4 School of Medicine, National Defense Medical Center, Neihu Dist., Taipei City, aiwan (R.O.C.); 5 Department of Physical Medicine and Rehabilitation, Tri-Service General Hospital, Neihu District, Taipei City, Taiwan(R.O.C.); 6 Management Office for Health Data, China Medical University Hospital, Taichung, Taiwan, Taichung, Taichung, Taiwan (R.O.C.); 7 Department of Neurology, China Medical University Hospital, Taichung, Taiwan, North District, Taichung, Taiwan (R.O.C.); 8 School of Medicine, China Medical University, Taichung, Taiwan, Taichung, Taichung, Taiwan (R.O.C.); 9 Graduate Institute of Clinical Medicine Science, College of Medicine, China Medical University, Taichung, Taiwan; Taichung, Taichung, Taiwan (R.O.C.); 10 Department of Health Services Administration, China Medical University, Taichung, Taiwan; Taichung, Taichung, Taiwan (R.O.C.); Osaka University Graduate School of Medicine, JAPAN

## Abstract

**Objective:**

This population-based cohort study investigated the risk of developing peripheral arterial occlusive disease (PAOD) in patients with Bell’s palsy.

**Methods:**

We used longitudinal claims data of health insurance of Taiwan to identify 5,152 patients with Bell’s palsy newly diagnosed in 2000–2010 and a control cohort of 20,608 patients without Bell’s palsy matched by propensity score. Incidence and hazard ratio (HR) of PAOD were assessed by the end of 2013.

**Results:**

The incidence of PAOD was approximately 1.5 times greater in the Bell’s palsy group than in the non-Bell’s palsy controls (7.75 vs. 4.99 per 1000 person-years). The Cox proportional hazards regression analysis measured adjusted HR was 1.54 (95% confidence interval (CI) = 1.35–1.76) for the Bell’s palsy group compared to the non-Bell’s palsy group, after adjusting for sex, age, occupation, income and comorbidities. Men were at higher risk of PAOD than women in the Bell’s palsy group, but not in the controls. The incidence of PAOD increased with age in both groups, but the Bell’s palsy group to control group HR of PAOD decreased as age increased. The systemic steroid treatment reduced 13% of PAOD hazard for Bell’s palsy patients, compared to those without the treatment, but not significant.

**Conclusions:**

Bell’s palsy appears to be associated with an increased risk of developing PAOD. Further pathophysiologic, histopathology and immunologic research is required to explore the underlying biologic mechanism.

## Introduction

Peripheral arterial occlusive disease (PAOD) is a circulation disease caused by atherosclerosis of peripheral vessels. It features with pain, pale, paresthesia and pulseless of limbs. The prevalence of PAOD ranges from 3.9%-26.2% among worldwide population.[1, 2] The increased risk of the disease has been associated with male gender, older age, smoking, diabetes, hypertension and dyslipidemia.[1, 3, 4] PAOD has attracted attentions recently for predicting morbidity of and mortality from cardiovascular diseases. A Californian study found a 2.8-fold increased mortality risk from cardiovascular diseases for patients with peripheral arterial disease after being tracked for 3 years.[5]

Bell’s palsy features with a sudden onset of unilateral facial paresis or paralysis. The symptom is caused by the inflammatory facial nerve, resulting from compression of the narrowest portion of the fallopian canal [6, 7]. The etiology is still unclear but viral and immunological hypothesis have been postulated to explain the potential pathophysiological mechanism. Latent virus reactivation from the geniculate ganglion may cause inflammation.[8] The most convincing evidence of pathogens is herpes simplex virus type 1 (HSV-1), which has been detected in endoneurial fluid of facial nerve in patients with Bell’s palsy [9, 10], followed by varicella-zoster virus (VZV) as the second common pathogen[11, 12]. Other pathogens such as CMV, EBV, mumps and rubella have been reported as well.[8] Bell’s palsy is more common between the ages of 15 to 45 years, with a lifetime risk of 1 in 60. [12, 13] It’s more prevalent in patients with diabetes and pregnant women.[14] It is important clinically to distinguish the disorder from stroke. Bell’s palsy is regarded as a benign disease with up to 70% of patients recovered completely, although about 30% of patients with the neurological sequelae remained.[14]

A recent study reported that patients with Bell’s palsy had a 2-fold increased risk of stroke.[15] To explore whether Bell’s palsy plays a pathologic role in other cardiovascular diseases, this study used claims data of the National Health Insurance (NHI) program of Taiwan to investigate the risk of developing PAOD in patients with and without Bell’s palsy.

## Materials and methods

### Data sources

The insurance program of Taiwan is a universal insurance program, reformed from all 13 insurance systems in 1995, providing comprehensive coverage for 99% of all residents. This study used the longitudinal health insurance database (LHID 2000), consisting of claims data of 1,000,000 insured individuals randomly selected from all 23 million insured population. Information on demographic data, inpatient and outpatient cares, date of clinic visit or hospitalization and prescriptions were available in the database for the period from 1996 to 2013. Diagnoses were coded in the format of the International Classification of Disease, Ninth Revision, Clinical Modification (ICD-9-CM). The authority had replaced the original identification numbers with surrogate numbers before the data were released to protect the people privacy. Our research was approved by the Research Ethics Committee of China Medical University and Hospital (CMUH104-REC2-115).

### Study population

From the LHID 2000 medical claims, we identified patients with Bell’s palsy (ICD-9-CM 351.0x), aged 20 years and older without the history of PAOD, newly diagnosed in 2000–2010, and included them in the Bell’s palsy group. The date with Bell’s palsy diagnosed was defined as the index date. Only patients who received systemic steroid treatment within 10 days after the date with Bell’s palsy diagnosed using anatomical therapeutic chemical (ATC) code (H02AB) were included in the present study. A non-Bell’s palsy controls were also selected from population without Bell’s palsy and PAOD, matched by propensity score to balance the two groups to augment their comparability.[16] We estimated the propensity score for every person using multivariate logistic regression, with Bell’s palsy as the dependent variable. We incorporated sex, age, year of index date, and comorbidities of diabetes mellitus (DM, ICD-9-CM 250), dyslipidemia (ICD-9-CM 272), hypertension (ICD-9-CM 401–405), CAD (ICD-9-CM 410–414), heart failure (ICD-9-CM 428), stroke (ICD-9-CM 430–438), chronic obstructive pulmonary disease (COPD; ICD-9-CM 492, 494, and 496), and asthma (ICD-9-CM 493), and herpes simplex virus (HSV; ICD-9-CM 054) into analysis as independent variables (all baseline characteristics). For each patient with Bell’s palsy, we chose 4 persons without Bell’s palsy with nearest propensity score by greedy algorithm. The index date of patient with Bell’s palsy was assigned for the matched cases. We also considered occupation status (white collar, blue collar and other) and income (<15000, 15000–30000, and >30000) for social economic status.

Our primary outcome was PAOD (ICD-9-CM 440.2, 440.3, 440.8, 440.9, 443, 444.22, 444.8, 447.8, and 447.9). Both Bell’s palsy and control groups were followed from the index date until they had the diagnosis of PAOD, or were censored for deaths or withdrawal from the insurance system, or the end of 2013.

### Statistics

The distribution of categorical variables (such as sex, age group, occupation, income, and history of comorbidity) were compared between Bell’s palsy and non-Bell’s palsy groups by the Chi-square test. The Student’s t-test was used to compare mean ages between the 2 cohorts. The incidence density rates of PAOD (per 1,000 person-years) were calculated for the Bell’s palsy and non-Bell’s palsy groups by potential risk factors, such as sex and age (20–44, 45–64, and ≥65 years old), and comorbidity (no/yes). The Kaplan-Meier method was used to draw cumulative incidence curves of PAOD for both groups and the curves were examined using log-rank test. Univariate and multivariate Cox proportional hazards regression models were used to assess hazard ratios (HRs) of PAOD for Bell’s palsy patients compared to the non-Bell’s palsy group by PAOD-associated factors. The multivariate model included variable of sex, age, occupation, income, and comorbidities (DM, dyslipidemia, hypertension, CAD, heart failure, stroke, COPD, asthma, and HSV). HRs of PAOD were evaluated by sex, age group, and comorbidity. We further evaluated whether the systemic steroid treatment could reduce the development of PAOD for Bell’s palsy patients.

We deemed a two-tailed p value less than 0·05 significant. SAS 9.4 software (SAS Institute, Cary, NC, USA) was performed data management and statistical analysis.

## Results

### Demographic characteristics

After matching participants in a 1:4 ratio by the propensity score, 5152 patients with Bell’s palsy and 20608 individuals without Bell’s palsy were included. Table 1 shows the study groups with and without Bell’s palsy were similar with respect to sex, age and all comorbidities. Patients with Bell’s palsy were more prevalent with white-collar and blue collar occupations and had higher income.

**Table 1:** Baseline demographic factors and comorbidity compared between Bell’s palsy group and non- Bell’s palsy group.

	Bell’s palsy				
	No		Yes		
	(N = 20608)		(N = 5152)		
Characteristics	n	%	n	%	p-value
Sex					0.93
Women	10010	48.6	2499	48.5	
Men	10598	51.4	2653	51.5	
Age, years					0.99
20–44	8122	39.4	2037	39.5	
45–64	8091	39.3	2020	39.2	
≥ 65	4395	21.3	1095	21.3	
Mean (SD)	49.9	(17.0)	50.1	(16.4)	0.39
Occupation					0.03
White collar	10698	51.9	2700	52.4	
Blue collar	7917	38.4	2016	39.1	
Other	1993	9.67	436	8.46	
Income					0.005
<15000	7443	36.1	1738	33.7	
15000–30000	9670	46.9	2526	49.0	
>30000	3495	17.0	888	17.2	
Comorbidity					
Diabetes	3607	17.5	903	17.5	0.98
Dyslipidemia	5823	28.3	1460	28.3	0.92
Hypertension	7695	37.3	1917	37.2	0.87
CAD	3890	18.9	981	19.0	0.80
Heart failure	536	2.60	153	2.97	0.16
Stroke	893	4.33	238	4.62	0.39
COPD	2580	12.5	653	12.7	0.78
Asthma	1673	8.12	425	8.25	0.78
HSV	411	1.99	111	2.15	0.50

Abbreviation: SD, standard deviation; CAD, coronary artery disease; COPD, chronic obstructive pulmonary disease; HSV, herpes simplex virus.

### Risk of developing PAOD

**Fig 1:**
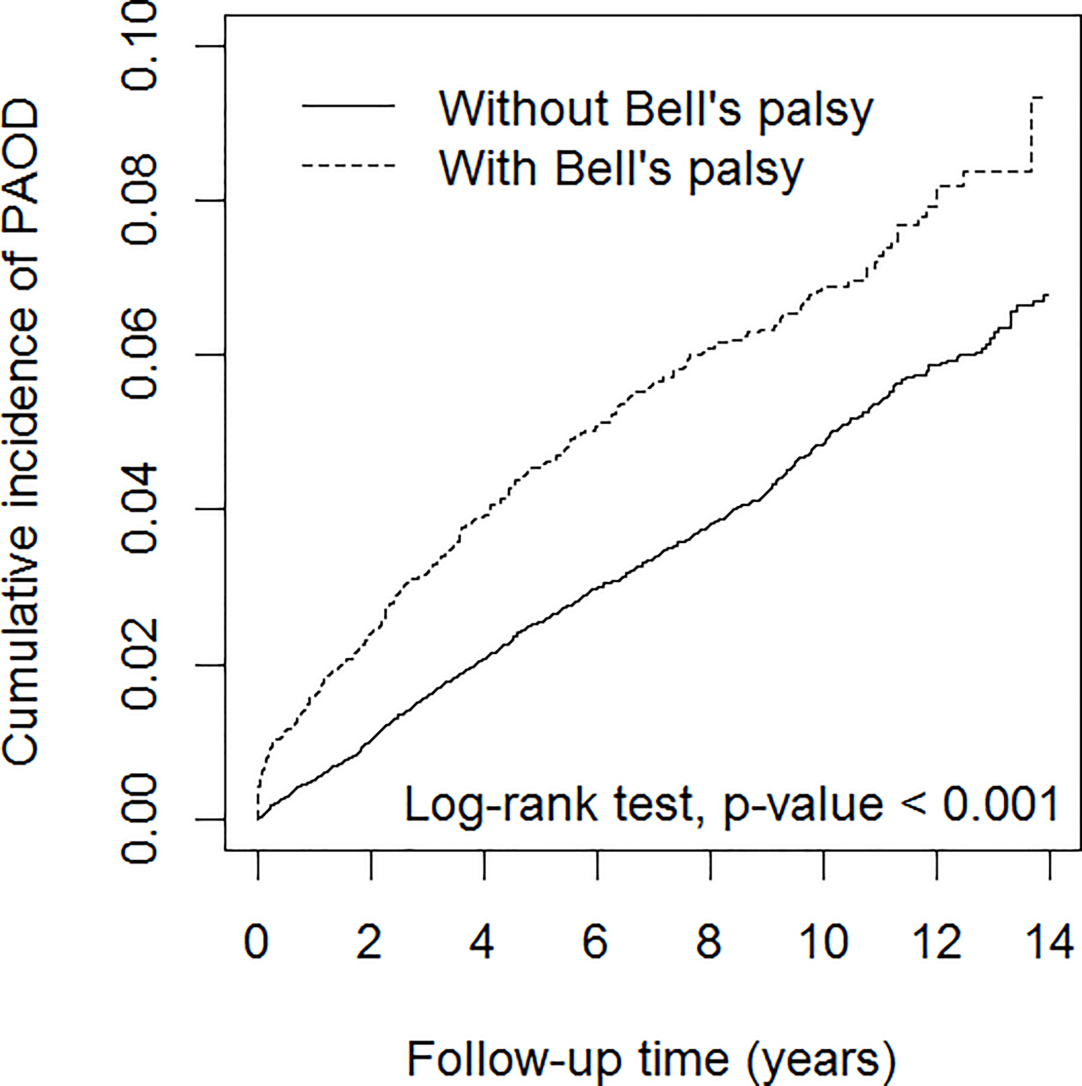
Cumulative incidence curves of peripheral arterial occlusive disease for groups with and without Bell’s palsy Abbreviation: PAOD, peripheral arterial occlusive disease.

During the mean follow-up period of 7.66 years, there were 303 patients diagnosed with PAOD in the Bell’s palsy group, with an incidence density rate of 7.75 per 1000 person-years, and 790 patients diagnosed with PAOD in the non-Bell’s palsy group, with an incidence density rate of 4.99 per 1000 person-years. Cumulative incidence curves of PAOD in patients with and without Bell’s palsy were shown in Fig 1. We found the cumulative incidence of PAOD was 2.8% higher in patients with Bell’s palsy than in subjects without Bell’s palsy (log-rank test, p < 0.001). After adjusting for sex, age, occupation, income, and comorbidity, Cox proportional hazards regression analysis showed that the Bell’s palsy group had an adjusted HR of 1.54 (95% confidence interval (CI) = 1.35–1.76) developing PAOD, compared to the non-Bell’s palsy group (Table 2). The incidence in the Bell’s palsy group was higher in men than in women, and the adjusted HR was greater for men than for women, compared with controls. The incidence increased with age, but the relative adjusted HR decreased with age. Comorbidity increased the risk of PAOD. For population without comorbidity, the Bell’s palsy group to the control group adjusted HR was 2.29 (95% CI = 1.67–3.16). Table 3 shows the incidence of PAOD was the highest in patients with comorbid heart failure based on the data by pooling both cohorts. However, the adjusted HR was the greatest for Bell’s palsy patients with hypertension.

**Table 2:** Incidence rates and Bell’s palsy group to controls hazard ratios of peripheral arterial occlusive disease measured by sex, age, and comorbidity.

	Without Bell’s palsy			With Bell’s palsy			HR (95% CI)	
Characteristics	Event no.	Person-years	IR	Event no.	Person-years	IR	Crude	Adjusted ^‡^
Overall	790	158254	4.99	303	39102	7.75	1.55 (1.36–1.77)	1.54 (1.35–1.76)
Sex								
Women	422	77639	5.44	143	19173	7.46	1.37 (1.13–1.66)	1.36 (1.12–1.64)
Men	368	80615	4.56	160	19928	8.03	1.75 (1.46–2.11)	1.74 (1.45–2.10)
Age, years								
20–44	74	67077	1.10	39	16748	2.33	2.13 (1.44–3.14)	2.10 (1.42–3.10)
45–64	342	62979	5.43	149	15210	9.80	1.80 (1.48–2.18)	1.79 (1.48–2.17)
≥ 65	374	28198	13.3	115	7144	16.1	1.21 (0.98–1.50)	1.21 (0.98–1.49)
Comorbidity status ^†^								
No	106	78064	1.36	59	19207	3.07	2.31 (1.68–3.18)	2.29 (1.67–3.16)
Yes	684	80190	8.53	244	19894	12.3	1.43 (1.24–1.66)	1.43 (1.24–1.66)

Abbreviation: IR, incidence density rate, per 1,000 person-years; HR, hazard ratio; CI, confidence interval.

^†^ Patients with any one of diabetes, dyslipidemia, hypertension, heart failure, stroke, COPD, asthma, and HSV were classified as the comorbidity group.

^‡^ Model mutually adjusting for sex, age (continuous), occupation, income, diabetes, dyslipidemia, hypertension, CAD, heart failure, stroke, COPD, asthma, and HSV.

**Table 3:** Cox model measured hazard ratios and 95% confidence interval of peripheral arterial occlusive disease by comorbidity.

Variable	Event no.	Person-years	IR	HR (95% CI)	
				Crude	Adjusted ^†^
Comorbidity					
DM					
No	714	167092	4.27	ref	ref
Yes	379	30263	12.5	2.90 (2.56–3.29)	1.34 (1.17–1.54)
Dyslipidemia					
No	558	146898	3.80	ref	ref
Yes	535	50458	10.6	2.77 (2.46–3.12)	1.26 (1.10–1.44)
Hypertension					
No	331	130082	2.54	ref	ref
Yes	762	67274	11.3	4.42 (3.89–5.03)	1.73 (1.47–2.04)
CAD					
No	636	164806	3.86	ref	ref
Yes	457	32549	14.0	3.61 (3.20–4.07)	1.48 (1.29–1.70)
Heart failure					
No	1033	193781	5.33	ref	ref
Yes	60	3574	17.0	3.04 (2.34–3.95)	0.95 (0.72–1.25)
Stroke					
No	1013	190701	5.31	ref	ref
Yes	80	9954	12.0	2.22 (1.76–2.78)	0.90 (0.71–1.14)
COPD					
No	856	176766	4.84	ref	ref
Yes	237	20589	11.5	2.34 (2.03–2.71)	1.06 (0.91–1.25)
Asthma					
No	955	183875	5.19	ref	ref
Yes	138	13480	10.2	1.93 (1.62–2.32)	1.08 (0.89–1.31)
HSV					
No	1071	193954	5.52	ref	ref
Yes	22	3401	6.47	1.15 (0.75–1.76)	1.22 (0.80–1.57)

Abbreviation: IR, incidence density rate, per 1,000 person-years; HR, hazard ratio; CI, confidence interval; CAD, coronary artery disease; COPD, chronic obstructive pulmonary disease; HSV, herpes simplex virus.

^†^ Model including Bell’s palsy, sex, age (continuous), occupation, income, diabetes mellitus, dyslipidemia, hypertension, CAD, heart failure, stroke, COPD, asthma, and HSV.

Further data analysis evaluating treatment effectiveness of systemic steroid showed that the non-users were at 1.4-fold greater PAOD risk than users (9.45 vs. 6.81 per per 1000 person-years) (Table 4). Bell’s palsy patients with systemic steroid treatment had the adjusted HR reduced to 0.87 (95% CI = 0.69–1.09), compared with non-users.

**Table 4:** Incidence rates and Cox method estimated hazard ratios of peripheral arterial occlusive disease by steroid therapy for Bell’s palsy.

	N	Event no.	Person-years	IR	HR (95% CI)			
					Crude		Adjusted^‡^	
Without Bell’s palsy	20608	790	158254	4.99	ref		ref	
With Bell’s palsy								
Systemic steroid non-users	1844	131	13857	9.45	1.89 (1.57–2.27)	ref	1.63 (1.35–1.96)	ref
Systemic steroid^†^ users	3308	172	25245	6.81	1.36 (1.15–1.61)	0.72 (0.58–0.91)	1.48 (1.26–1.75)	0.87 (0.69–1.09)

Abbreviation: IR, incidence density rate, per 1,000 person-years; HR, hazard ratio; CI, confidence interval.

^†^ Systemic steroid including oral steroid and IV steroid

^‡^ Model adjusting for sex, age (continuous), occupation, income, diabetes, dyslipidemia, hypertension, CAD, heart failure, stroke, COPD, asthma, and HSV.

## Discussion

To the best of our knowledge, this is the first retrospective cohort study using population data to investigate the risk of developing PAOD following the Bell’s palsy occurrence. We demonstrated a 54% increased hazard of subsequent PAOD in Bell’s palsy cohort compared with controls. Besides, the effect is more prominent in young patients and patients without comorbidity.

Several studies have investigated mechanisms to explain the association between PAOD and Bell’s palsy. Bell’s palsy is acknowledged widely as reactivation of latency virus of geniculate ganglion, with HSV-1 and VZV are known more common pathogens [11, 17]. Virus infection could lead to inflammation and subsequently result in atherosclerosis [18]. Animal study have linked Marek’s disease and herpesvirus to atherosclerosis in both hypercholeterolemic and normocholeterolemic chickens.[19] In situ hybridization of human aortic wall, herpesvirus (included HSV-1, EBV, and CMV) was found to be associated with atherosclerotic plaque.[20] Through pathophysiologic studies by the artery biopsy of human, HSV was found in patients with an early atherosclerotic lesion[18, 21] and in patients prevalent with temporal arteritis.[22] Furthermore, pathologic and virological evidence show that VZV DNA and antigen are compatible with ischemia and infarction in stroke cases.[23] The immunohistochemistry evidence shows that VZV can emerge in vascular adventitia early and in media and intimal later. Thus, the latent virus spreads transaxonally from ganglion to arteries, leading to the development of arteriosclerosis.[24] Epidemiologic studies have shown dose-response relationships between seropositive infections, including HSV-1 (primarily IgG), and progression of atherosclerosis and CAD.[18, 25, 26] Furthermore, in a population-based study, Lee et al. found an increased risk of stroke in patients with Bell’s palsy during a 3-year follow-up period.[15]

However, the diagnosis accuracy is a common concern of using medical registry database, especially for Bell’s palsy and stroke because the sudden onset of unilateral facial palsy of both disorders may receive more attention from physicians. In this aspect, our study outcome of increased risk of PAOD in Bell’s palsy cohort supported that Bell’s palsy played the pathologic role in cardiovascular disease risk and contained vascular risk components in this idiopathic neurologic inflammation disease.

Our study showed that the PAOD risk in patients with Bell’s palsy increased for those with older ages, diabetes, hypertension and dyslipidemia. Our findings are compatible with previous study.[1, 3, 4, 27] In considering of gender, men are generally considered at a higher risk than women for PAOD.[3] However, recent epidemiologic studies showed conflicting results.[28, 29] A life line screening program enrolled about 200,000 citizens in the US showed that women were more prevalent with peripheral artery disease than men, but the conventional CVD risk could not explain this gender difference.[27] A systemic review based on 34 studies showed that the prevalence of PAOD is higher in women than in men in low- and middle- income countries, but no significant difference between women and men in high income countries.[30] Our study findings in gender difference showed that the incident PAOD was slightly higher in men than in women in the Bell’s palsy group, which is compatible with findings in high income countries. However, the incident PAOD was lower in men than in women in the non-Bell’s palsy group. Therefore, the Bell’s palsy group to the non-Bell’s palsy group HR was greater for men than for women.

It is interesting to note that the incidence of PAOD increased with age in both cohorts with and without Bell’s palsy. But, the Bell’s palsy group to non-Bell’s palsy group HR was greater in younger than in the elderly, indicating the impact of Bell’s palsy is relatively greater for younger Bell’s palsy patients. On the other hand, the corresponding relative adjusted HR for the elderly was 1.21 (95% CI = 0.98–1.49). Our data analysis by comorbidity showed that patients with cardiovascular diseases and diabetes have increased PAOD risk in both Bell’s palsy and control cohorts. Cardiovascular diseases and diabetes were more prevalent in the elderly in both cohorts. This is why the relative adjusted HR for the elderly was lower than that for the younger. The effect of Bell’s palsy is particularly prominent in the youngest subgroup and the subgroup without comorbidity. These findings support that Bell’s palsy is an independent risk factor for PAOD.

It is important to note the treatment effectiveness of systemic steroid treatment for Bell’s palsy patients. A recent study found a beneficial effect for Bell’s palsy patients in reducing stroke risk.^15^ Our further data analysis compared the PAOD risk between Bell’s palsy patients with and without systemic steroid treatment. Results showed that Bell’s palsy patients with and without systemic steroid treatment consistently had a significantly higher risk of PAOD than in subjects without Bell’s palsy. Our result also revealed that the systemic steroid treatment had a moderate effect in reducing the risk of the long term PAOD risk for patient with Bell’s palsy, but not significant. In an earlier study, Gilden reported that Bell’s palsy treatment only supported a short course of prednisone medication within 2 to 14 days after the onset of symptoms.[10] A short course of steroid medication might not able to alter virus vasculopathy or systemic inflammation condition in the long term follow-up. Our study highlights clinicians to remind the risk of PAOD in patients with Bell’s palsy. Further evaluation on preventive medication as anti-plate agents, antiviral medication, or even long-term steroid treatment is warranted.

Our study had some potential limitations. First, diagnosis accuracy was always concerned in using medical registry database. However, in addition to verification coding by licensed medical record technician after physician competing discharge chart, routine sampling of charts to cross-over exam by specialists at the NHI Bureau to ensure the validity and accuracy of disease coding. Second, there’s some meaningful information not available in NHIRD, such as smoking, daily activity and obesity. However, we minimized the effect of these confounding factors using COPD, CAD and stroke as proxy in the data analysis. Third, information on histopathology of diseases is unavailable in the claims data, the histopathology relationship could not confirmed between Bell's palsy and PAOD. Finally, the patient with asymptomatic PAOD may not seek medical assistance and may not identified in our study. Therefore, it led to underestimate incidence of PAOD in both Bell’s palsy and control cohorts. Despite these limitations, our nationwide population-based study using information from the compulsory health insurance database over a 14-year period provided a sufficient sample size to investigate the association between Bell’s palsy and PAOD risk.

## Conclusion

The findings from this large-scale follow-up study could conclude that Bell’s palsy was associated with 49% increased risk of developing PAOD. Low-dose maintenance systemic steroid therapy for a longer period would be an option to reduce the risk and deserve study. Further research focused on pathophysiologic, histopathology and immunologic issues is also warranted to clarify the underlying biologic mechanism.

## References

[1] Joosten MM, Pai JK, Bertoia ML, Rimm EB, Spiegelman D, Mittleman MA, et al Associations between conventional cardiovascular risk factors and risk of peripheral artery disease in men. Jama. 2012;308(16):1660–7. doi: 10.1001/jama.2012.13415 23093164 10.1001/jama.2012.13415 PMC3733106

[2] Alzamora MT, Fores R, Baena-Diez JM, Pera G, Toran P, Sorribes M, et al The peripheral arterial disease study (PERART/ARTPER): prevalence and risk factors in the general population. BMC public health. 2010;10:38 doi: 10.1186/1471-2458-10-38 ; PubMed Central PMCID: PMC2835682. 20529387 10.1186/1471-2458-10-38 PMC2835682

[3] Novo S. Classification, epidemiology, risk factors, and natural history of peripheral arterial disease. Diabetes, Obesity and Metabolism. 2002;4(s2):S1–S6. 10.1046/j.1463-1326.2002.0040s20s1.x 12180352

[4] Selvin E, Erlinger TP. Prevalence of and risk factors for peripheral arterial disease in the United States: results from the National Health and Nutrition Examination Survey, 1999–2000. Circulation. 2004;110(6):738–43. doi: 10.1161/01.CIR.0000137913.26087.F0 . 15262830 10.1161/01.CIR.0000137913.26087.F0

[5] Criqui MH, Ninomiya JK, Wingard DL, Ji M, Fronek A. Progression of peripheral arterial disease predicts cardiovascular disease morbidity and mortality. Journal of the American College of Cardiology. 2008;52(21):1736–42. doi: 10.1016/j.jacc.2008.07.060 ; PubMed Central PMCID: PMC2871035. 19007695 10.1016/j.jacc.2008.07.060 PMC2871035

[6] Jackson CG, von Doersten PG. The facial nerve: current trends in diagnosis, treatment, and rehabilitation. Medical Clinics of North America. 1999;83(1):179–95. 9927969 10.1016/s0025-7125(05)70096-1

[7] Zandian A, Osiro S, Hudson R, Ali IM, Matusz P, Tubbs SR, et al The neurologist's dilemma: a comprehensive clinical review of Bell's palsy, with emphasis on current management trends. Medical science monitor: international medical journal of experimental and clinical research. 2014;20:83–90. doi: 10.12659/MSM.889876 ; PubMed Central PMCID: PMC3907546. 24441932 10.12659/MSM.889876 PMC3907546

[8] Greco A, Gallo A, Fusconi M, Marinelli C, Macri GF, de Vincentiis M. Bell's palsy and autoimmunity. Autoimmunity reviews. 2012;12(2):323–8. doi: 10.1016/j.autrev.2012.05.008 . 22684016 10.1016/j.autrev.2012.05.008

[9] Murakami S, Mizobuchi M, Nakashiro Y, Doi T, Hato N, Yanagihara N. Bell palsy and herpes simplex virus: identification of viral DNA in endoneurial fluid and muscle. Annals of internal medicine. 1996;124(1_Part_1):27–30. 7503474 10.7326/0003-4819-124-1_part_1-199601010-00005

[10] Gilden DH. Bell's palsy. New England Journal of Medicine. 2004;351(13):1323–31. doi: 10.1056/NEJMcp041120 15385659 10.1056/NEJMcp041120

[11] Furuta Y, Ohtani F, Kawabata H, Fukuda S, Bergström T. High prevalence of varicella-zoster virus reactivation in herpes simplex virus-seronegative patients with acute peripheral facial palsy. Clinical infectious diseases. 2000;30(3):529–33. doi: 10.1086/313721 10722439 10.1086/313721

[12] Holland NJ, Weiner GM. Recent developments in Bell's palsy. BMJ: British Medical Journal. 2004;329(7465):553 doi: 10.1136/bmj.329.7465.553 15345630 10.1136/bmj.329.7465.553 PMC516110

[13] Holland J, Bernstein JM. Bell's palsy. Clinical evidence. 2008;2008.

[14] Peitersen E. Bell's palsy: the spontaneous course of 2,500 peripheral facial nerve palsies of different etiologies. Acta Oto-Laryngologica. 2002;122(7):4–30. 12482166

[15] Lee CC, Su YC, Chien SH, Ho HC, Hung SK, Lee MS, et al Increased stroke risk in Bell's palsy patients without steroid treatment. European journal of neurology. 2013;20(4):616–22. doi: 10.1111/j.1468-1331.2012.03765.x . 22672698 10.1111/j.1468-1331.2012.03765.x

[16] Stürmer T, Wyss R, Glynn RJ, Brookhart MA. Propensity scores for confounder adjustment when assessing the effects of medical interventions using nonexperimental study designs. Journal of internal medicine. 2014;275(6):570–80. doi: 10.1111/joim.12197 24520806 10.1111/joim.12197 PMC4037382

[17] Furuta Y, Takasu T, Sato K, Fukuda S, Inuyama Y, Nagashima K. Latent herpes simplex virus type 1 in human geniculate ganglia. Acta neuropathologica. 1992;84(1):39–44. 1323906 10.1007/BF00427213

[18] Elkind V, Mitchell S. Infectious burden: a new risk factor and treatment target for atherosclerosis. Infectious Disorders-Drug Targets (Formerly Current Drug Targets-Infectious Disorders). 2010;10(2):84–90. 10.2174/187152610790963519 PMC2891124 20166973

[19] Fabricant C, Fabricant J, Minick C, Litrenta M, editors. Herpesvirus-induced atherosclerosis in chickens. Federation proceedings; 1983. 6840298

[20] Shi Y, Tokunaga O. Herpesvirus (HSV‐1, EBV and CMV) infections in atherosclerotic compared with non‐atherosclerotic aortic tissue. Pathology international. 2002;52(1):31–9. 11940204 10.1046/j.1440-1827.2002.01312.x

[21] Benditt EP, Barrett T, McDougall JK. Viruses in the etiology of atherosclerosis. Proceedings of the National Academy of Sciences. 1983;80(20):6386–9. 10.1073/pnas.80.20.6386 PMC394302 6312457

[22] Powers JF, Bedri S, Hussein S, Salomon RN, Tischler AS. High Prevalence of Herpes Simplex Virus DNA in Temporal Arteritis Biopsy Specimens. American Journal of Clinical Pathology. 2005;123(2):261–4. doi: 10.1309/2996tt2ctltkn0kt 15842052 10.1309/2996tt2ctltkn0kt

[23] Nagel M, Mahalingam R, Cohrs R, Gilden D. Virus vasculopathy and stroke: an under-recognized cause and treatment target. Infectious Disorders-Drug Targets (Formerly Current Drug Targets-Infectious Disorders). 2010;10(2):105–11. 10.2174/187152610790963537 PMC2909030 20166970

[24] Nagel M, Traktinskiy I, Azarkh Y, Kleinschmidt-DeMasters B, Hedley-Whyte T, Russman A, et al Varicella zoster virus vasculopathy Analysis of virus-infected arteries. Neurology. 2011;77(4):364–70. doi: 10.1212/WNL.0b013e3182267bfa 21753174 10.1212/WNL.0b013e3182267bfa PMC3140801

[25] Espinola-Klein C, Rupprecht H-J, Blankenberg S, Bickel C, Kopp H, Victor A, et al Impact of infectious burden on progression of carotid atherosclerosis. Stroke. 2002;33(11):2581–6. 12411646 10.1161/01.str.0000034789.82859.a4

[26] Zhu J, Quyyumi AA, Norman JE, Csako G, Waclawiw MA, Shearer GM, et al Effects of total pathogen burden on coronary artery disease risk and C-reactive protein levels. The American journal of cardiology. 2000;85(2):140–6. 10955367 10.1016/s0002-9149(99)00653-0

[27] Hiramoto JS, Katz R, Weisman S, Conte M. Gender-specific risk factors for peripheral artery disease in a voluntary screening population. Journal of the American Heart Association. 2014;3(2):e000651 doi: 10.1161/JAHA.113.000651 ; PubMed Central PMCID: PMC4187488. 24627420 10.1161/JAHA.113.000651 PMC4187488

[28] Brevetti G, Bucur R, Balbarini A, Melillo E, Novo S, Muratori I, et al Women and peripheral arterial disease: same disease, different issues. J Cardiovasc Med (Hagerstown). 2008;9(4):382–8. doi: 10.2459/JCM.0b013e3282f03b90 18334893 10.2459/JCM.0b013e3282f03b90

[29] Hirsch AT, Allison MA, Gomes AS, Corriere MA, Duval S, Ershow AG, et al A call to action: women and peripheral artery disease: a scientific statement from the American Heart Association. Circulation. 2012;125(11):1449–72. doi: 10.1161/CIR.0b013e31824c39ba Epub 2012 Feb 15. 22343782 10.1161/CIR.0b013e31824c39ba

[30] Fowkes FGR, Rudan D, Rudan I, Aboyans V, Denenberg JO, McDermott MM, et al Comparison of global estimates of prevalence and risk factors for peripheral artery disease in 2000 and 2010: a systematic review and analysis. The Lancet. 2013;382(9901):1329–40. doi: 10.1016/s0140-6736(13)61249-0 10.1016/S0140-6736(13)61249-0 23915883

